# Air pollution: Impact and prevention

**DOI:** 10.1111/j.1440-1843.2012.02213.x

**Published:** 2012-09-21

**Authors:** MARTHA PATRICIA SIERRA-VARGAS, LUIS M TERAN

**Affiliations:** 1 National Institute for Respiratory Diseases ‘Ismael Cosío Villegas’México; 2 Biomedicine in the Post-Genomic EraHuitzilac, Morelos, Mexico

**Keywords:** air pollution, health impact, prevention, respiratory disease

## Abstract

Air pollution is becoming a major health problem that affects millions of people worldwide. In support of this observation, the World Health Organization estimates that every year, 2.4 million people die because of the effects of air pollution on health. Mitigation strategies such as changes in diesel engine technology could result in fewer premature mortalities, as suggested by the US Environmental Protection Agency. This review: (i) discusses the impact of air pollution on respiratory disease; (ii) provides evidence that reducing air pollution may have a positive impact on the prevention of disease; and (iii) demonstrates the impact concerted polices may have on population health when governments take actions to reduce air pollution.

## INTRODUCTION

Environmental pollution has been a matter of concern for many years. The Mellon Institute of Pittsburgh, PA, USA, sponsored the first broad scientific study of smoke abatement, which resulted in legislation designed to decrease the effects of smoke.^1^ It is now well known that environmental contamination impacts on health; the World Health Organization estimates that every year, 2.4 million people die from causes associated with air pollution. It is increasingly recognized that implementation of strategies to reduce pollution can have substantial health benefits. For example, the Environmental Protection Agency proposed that the implementation of measures to reduce emissions from diesel engines could result in 12 000 fewer mortalities and prevent 15 000 heart attacks and 8900 hospital admissions in the United States each year.^2^ The aim of this review is to provide information on the impact of pollution on respiratory health, as well as to discuss strategies for reducing air pollution, as proposed in a number of clinical reports. Particulate matter (PM) and ozone (O_3_) pollution are major causes of concern in the community.

## PARTICULATE MATTER (PM)

PM is a complex mixture of solid and liquid particles suspended in air that is released into the atmosphere when coal, gasoline, diesel fuels and wood are burned. It is also produced by chemical reactions of nitrogen oxides and organic compounds that occur in the environment. Vegetation and livestock are also sources of PM. In big cities, production of PM is attributed to cars, trucks and coal-fired power plants.

The health effects of PM depend on several factors, including the size and composition of the particles, the level and duration of exposure, and the gender, age and sensitivity of the exposed individual. Symptoms of exposure may include persistent cough, sore throat, burning eyes and chest tightness. PM may also trigger asthma or lead to premature death, particularly in elderly individuals with pre-existing disease.^3,4^ In addition, people who are active outdoors are at higher risk, as physical activity increases the amounts of PM penetrating into the airways. People with disease (e.g. diabetes mellitus, malnutrition) are also at increased risk.^5–7^ A comprehensive review on diesel PM by Ristovski *et al*. was published in an earlier issue of this review series on air pollution and lung disease.^8^

## OZONE (O_3_)

O_3_ is mainly formed by the interaction of ultraviolet light with both nitrogen oxides and organic compounds. O_3_ exhibits potent anti-oxidant properties and induces alterations in the airways that depend on concentration and the duration of exposure.

## EFFECTS ON RESPIRATORY HEALTH

**Figure 1:**
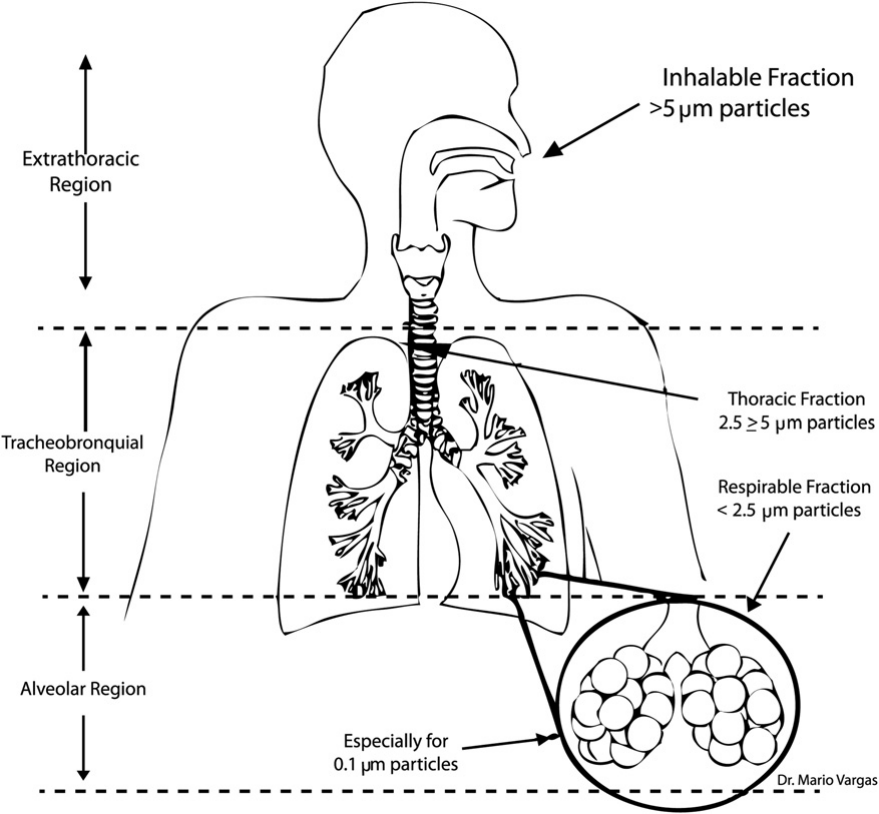
Regional deposition of particles in the human respiratory tract.

The airways are a point of entry for pollutants, which in turn may cause lung disease. For example, PM may be deposited into any of the three respiratory compartments: the extrathoracic, tracheobronchial and alveolar regions.^9^ PM > 10 µm in diameter (coarse particles) is deposited in the extrathoracic region, PM with a diameter between 5 and 10 µm is deposited in the tracheobronchial region and particles <2.5 µm in diameter (fine particles) are deposited in the alveolar region (Fig. 1, Table 1).^10^ For particles between 3 and 5 µm in diameter, the total deposition fraction is greater for women than for men.^11^ The potential health effects of greatest concern are associated with particles that penetrate to the tracheobronchial and alveolar regions.^12^ The deposition rate may also be increased in individuals with pre-existing respiratory disease, as compared with healthy individuals.^13^ It has been suggested that particles ≤0.1 µm in diameter (ultrafine particles) are more toxic than larger particles as they may cover a greater area of the alveolus. One host defence mechanism is phagocytosis of ultrafine particles by alveolar macrophages (Fig. 2). However, due to their small size, ultrafine particles overwhelm macrophage phagocytosis, resulting in increased penetration, which causes deleterious effects in other organs (e.g. brain, heart, bone marrow, etc.).^14,15^

**Table 1:** Environmental Protection Agency (EPA) terminology for particle sizes

EPA description	Particle size
Supercoarse	d_pa_ > 10 µm
Coarse	2.5 < d_pa_ ≤ 10 µm
Fine	0.1 < d_pa_ ≤ 2.5 µm
Ultrafine	d_pa_ ≤ 0.1 µm

d_pa_, aerodynamic particle diameter.

Data taken from http://www.epa.gov/apti/bces/module3/category/category.htm

**2:**
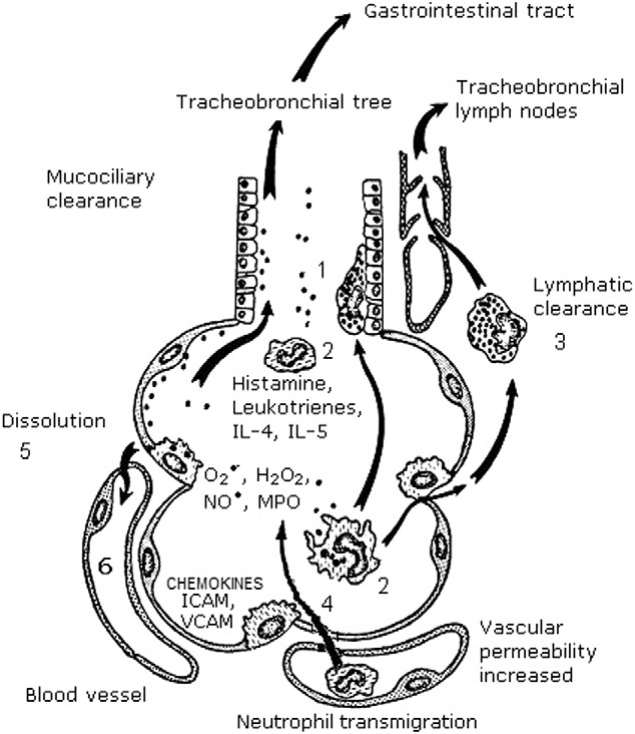
Alveolar deposition of particles and cell activation. Particles deposited in the bronchoalveolar region may be trapped and are cleared by the mucus layer (1); particles phagocytosed by alveolar macrophages follow the lymphatic clearance pathway, can impair phagocytosis and trigger the release of inflammatory mediators (2, 3) and neutrophil chemotactic factors, which in turn results in the release of reactive oxygen and nitrogen species (4). Furthermore, soluble particle components (e.g. metals) can cross the epithelial barrier and be distributed to other organs where they can cause adverse effects (5). ICAM, intercellular adhesion molecule; IL, interleukin; MPO, myeloperoxidase; VCAM, vascular cell adhesion molecule (6).

Toxicological studies have demonstrated the translocation of particles from the olfactory mucosa via axons to the olfactory bulb of the brain.^16,17^ Indoor activities in the home that result in the generation of particles include cooking (in ovens, toasting, frying, barbecuing), cleaning (dusting, sweeping, vacuuming) and the movement of people. Ozkaynak *et al*. reported that cooking resulted in the generation of 4.1 ± 1.6 mg/min of PM_10_, with the fine fraction contributing 40% of the total PM.^18^ Once PM enters the body, it affects different organ systems.

The source and composition of particles determine their toxicity,^19,20^ but size is a major factor determining toxicity in the lungs due to the generation of reactive oxygen and reactive nitrogen species. Particle size may also physically hinder macrophage clearance,^21^ thereby increasing toxicity. In general, particles exacerbate acute and pre-existing respiratory diseases, including viral infections, asthma, bronchitis and chronic respiratory disease.^22–24^ Many transition metals present on particles serve as catalysts for a Fenton-like reaction that initiates the production of reactive oxygen and reactive nitrogen species, resulting in an inflammatory response. Using electron microscopy, Brauer *et al*.^25^ showed significantly higher particle concentrations in the lungs, at autopsy of Mexican females who had never smoked, as compared with control Vancouver residents. Interestingly, Budinger *et al*.^26^ reported that inhalation of PM_2.5_ was sufficient to activate coagulation and inhibit fibrinolysis. Traffic particles appear to be more strongly associated with these effects,^27^ as they are rich in elemental carbon, which can cause an increase in respiratory symptoms in children^28,29^ and women living in urban areas.^30^

It is now well established that exposure to O_3_ impairs lung function. In healthy individuals, O_3_ causes reductions in vital capacity, forced expiratory volume in 1 s and lung resistance. The effects of O_3_ exposure increase with physical exercise. Patients with respiratory diseases are more susceptible to the effects of O_3_. Under conditions of oxidizing air pollution, as in summer, O_3_ exposure may lead to asthma exacerbations.

## ALLERGIC DISEASES

Allergic diseases such as asthma and allergic rhinitis are very common in children and young adults. In most cases, asthma in these groups of patients is characterized by increased synthesis of immunoglobulin E against common allergens.^31^ Exposure of these patients to specific aeroallergens such as pollens leads to a series of immunological changes culminating in the symptoms of asthma. It is now well established that increased air pollution affects pollen production, which in turn impacts negatively on the prevalence and severity of allergic asthma.

Diesel exhaust contains numerous pollutants and polycyclic aromatic hydrocarbons, which enhance allergenicity and asthma symptoms by acting in synergy with allergens. Experimental studies conducted by Muranaka *et al*.^32^ showed that diesel-exhaust particles act as an adjuvant for immunoglobulin E production in response to specific allergens (ovalbumin or Japanese Cedar pollen). Moreover, inhalation of diesel-exhaust particle leads to a typical asthma phenotype, characterized by pulmonary inflammation and airway hyperresponsiveness.^33,34^ It has been proposed that when diesel-exhaust particles are engulfed by macrophages, a Th2-type inflammatory response is induced, whereas diesel-exhaust particles that are not engulfed produce a Th1-type inflammatory response.^35–37^

High carbon dioxide concentrations in the environment increase both pollen production and the allergenicity of pollen. Indeed, Singer *et al*.^38^ showed that high concentrations of carbon dioxide enhanced the production of Amb a 1, an allergenic protein in ragweed pollen, while Ziska *et al*.^39^ reported that in urban locations where carbon dioxide concentrations are higher, ragweed produces greater amounts of pollen (which contains the Amb allergen) than it does in rural locations. The enhanced allergenicity of pollen may be explained by the synergistic association between allergen-loaded pollen debris and aromatic hydrocarbons contained in fine particles.^40^

On the other hand, traffic-related pollutants (nitrogen dioxide, O_3_) can trigger the release of allergens from pollen granules, leading to an increase in the concentration of airborne pollen allergens.^41^ For example, Dutch children attending schools that were within 400 m of a major roadway showed increased sensitization to outdoor allergens; the relationship between symptoms and traffic-related pollution was observed mainly in children who were sensitive to allergens.^42^ Similarly, increased levels of O_3_ and PM_2.5_ in summer were found to be associated with a higher prevalence of respiratory allergy symptoms in US children living in urban areas.^43^ D'Amato *et al*.^44^ hypothesize that air pollutants: (i) allow easier penetration of pollen allergens into the airways; (ii) increase the release of antigens from pollen grains, thereby leading to allergic responses; and (iii) absorb pollen grains, leading to prolonged retention of pollen grains in the body. A recent prospective birth cohort study involving over 2000 children showed that exposure to ambient PM increased the risk of atopic diseases.^45^

## MECHANISMS OF LUNG DAMAGE

### Oxidative stress

Oxidative stress plays a central role in the mechanisms by which air pollutants damage human health. In addition, reactive nitrogen species are generated in the lungs following exposure to particles. Nitric oxide released by inflammatory cells reacts with superoxide anion radicals to form peroxynitrite, which then initiates the nitration of tyrosine residues on proteins. These changes contribute to the progression of disease.^46,47^ On the other hand, the endogenous pool of H_2_O_2_ reacts with some enzymes such as myeloperoxidase to produce highly reactive metabolites (hypochlorous acid).^47,48^ Vujovic *et al*. reported an increase in malondialdehyde concentrations, whereas there was a reduction in superoxide dismutase activity (anti-oxidant defence) in children exposed to air pollution.^49^ Similarly, increased plasma levels of thiobarbituric acid reactive substances have been associated with exposure to black carbon and PM_2.5_.^50^ Individuals living in a polluted environment also showed increased plasma levels of thiobarbituric acid reactive substances.^52^

In animals, intratracheal instillation of PM causes a significant increase in serum levels of cytokines such as interleukin-6.^53^ Human macrophages exposed to particles, release a range of cytokines, including tumour necrosis factor-α, interleukin-6, interleukin-1β, macrophage inflammatory protein-1-α and granulocyte macrophage-colony stimulating factor.^54^ These cytokines activate nuclear factor kappa B and/or activator protein 1.^55,56^ Ultrafine black carbon is also involved in the activation of nuclear factor kappa B via protein kinase C.^57,58^ Metals contained in PM can induce a series of redox reactions causing oxidative DNA damage.

We have shown that exposure to O_3_ results in the release of increased levels of growth-related oncogene-α into the airway lining fluid in normal subjects.^59^ Interestingly, in a separate study, we demonstrated that neutrophils from asthmatic patients exposed to fine particles (PM_2.5_) generated reactive oxygen species.^60^ There is also a relationship between air pollution and cancer; pollutants may increase the risk of cancer through the formation of reactive oxygen species, especially hydroxyl and superoxide anion radicals, which may induce oxidative damage to cellular membrane lipids, protein enzymes and DNA.^61^

## PREVENTION OF AIR POLLUTION

### Clinical studies

It is well understood that pollution has a profound effect on health; therefore, reduction of pollution has a positive effect on health, particularly the health of susceptible individuals. The first population-based study that showed significant improvements in life expectancy in relation to reductions in PM_2.5_ concentrations was conducted in the United States,^62^ and showed a clear relationship between reduction in fine-particle concentrations and life expectancy. This observation was confirmed in a cohort study of Swiss adults, which demonstrated that decreases in ambient PM_10_ levels were associated with reductions in respiratory symptoms.^63^

**Figure 3:**
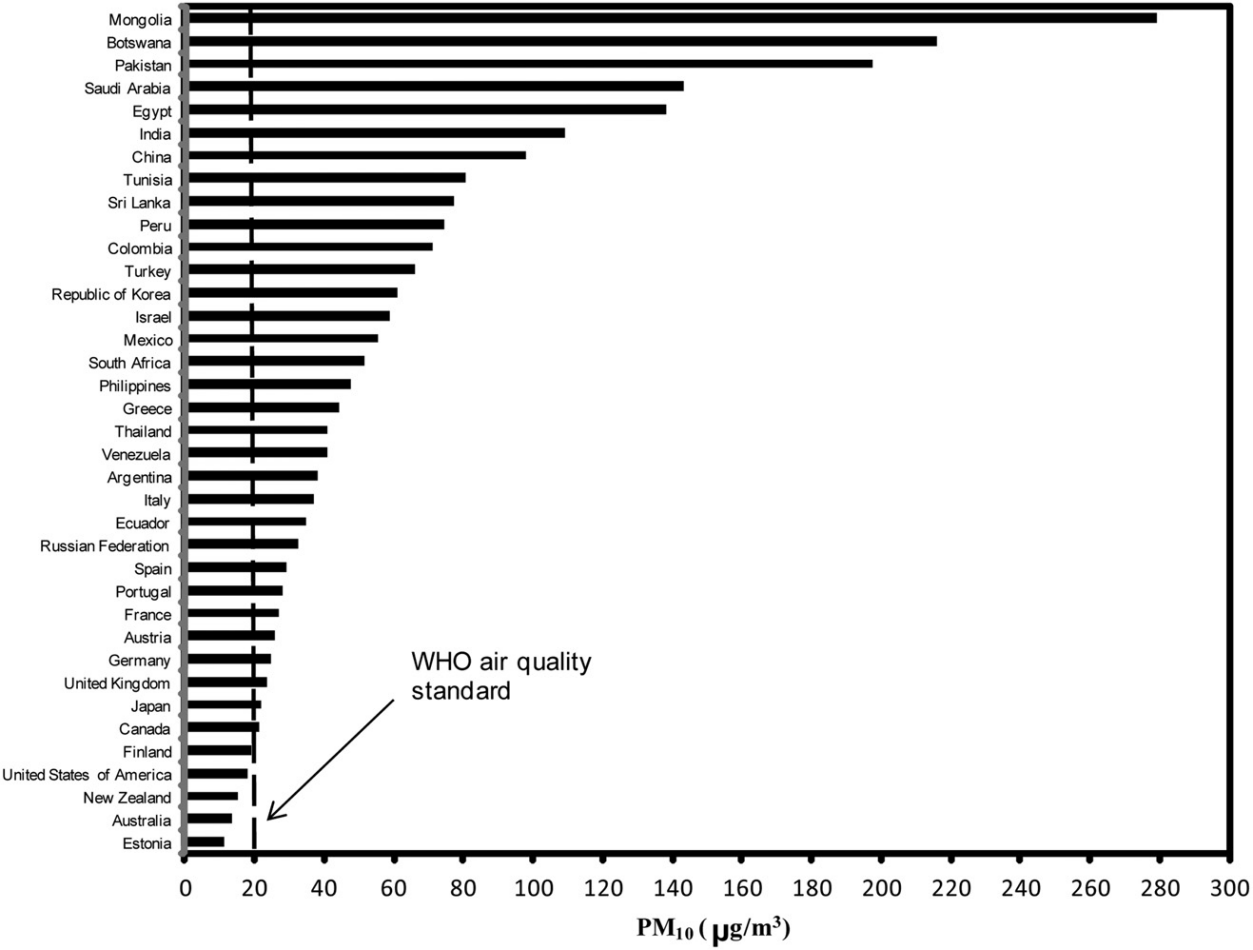
Pollution in 37 cities selected from 91 countries, as reported by the World Health Organization (WHO). Particulate matter (PM)_10_ levels >20 µg/m^3^ may pose health risks (data taken from http://apps.who.int/ghodata/?vid=4201).

Reductions in the levels of air pollution can be achieved in many ways, and governments can play a key role. Figure 3 shows PM_10_ levels in some of the most polluted countries. For example, during the 2008 Olympic Games, the Chinese government was able to control air pollution.^64^ This resulted in a 41.6% decrease in the average number of outpatient visits for asthma during the Olympics, as compared with before the games started. A separate study of 36 fourth-grade Beijing children, before, during and after the Beijing Olympics, showed that fractional exhaled nitric oxide (FeNO) levels were significantly lower during the period of the Olympics and increased by 16.6% in the first hours after exposure, suggesting that rapid inflammatory changes took place.^65^

In rural Mexico, a randomized trial of properly vented wood-burning cooking stoves versus open fires showed reductions in the longitudinal decline in forced expiratory volume in 1 s and improvements in respiratory symptoms, when proper cooking stoves were used.^66^ The use of improved cooking stoves has also been found to halve the exposure to carbon monoxide and resulted in a lower rate of diagnosis of pneumonia.^67^

Evidence is accumulating that polymorphisms in several genes involved in oxidative stress play an important role in susceptibility to O_3_.^68–70^ These genes include those coding for phase II enzymes, including glucuronosyl transferases, glutathione *S*-transferases (GST), NAD(P)H:quinone oxidoreductases and *N*-acetyltransferases, all of which mediate the detoxification and elimination of toxic products.

GSTM1 and GSTP1 have been the most frequently studied enzymes. A study of Mexican children exposed to high concentrations of O_3_ showed an association between polymorphisms in the oxidative stress-related GSTM1 gene and the development of asthma.^68^ Interestingly, children with the GSTM1-null genotype were more susceptible to the effects of O_3_. However, taking supplements containing the anti-oxidant vitamins C and E conferred protection against O_3_ exposure.^68^ Similarly, sulforaphane, a potent inducer of phase II enzymes, was also shown to enhance enzyme expression and downregulate inflammatory responses in human bronchial epithelial cells;^69^ sulforaphane increased GSTM1 and NAD(P)H:quinone oxidoreductase 1 expression, as well as GST activity while decreasing cytokine production. More recently, long-term supplementation with D-α-tocopheryl acetate, a natural vitamin E anti-oxidant, was shown to inhibit oxidant stress in the airways of mild atopic asthmatics exposed to segmental allergen challenge; improvements in allergic inflammation and bronchial hyperreactivity were observed.^70^ Studies of additional genes involved in the oxidative stress response have been reviewed previously.^71^

## AIR POLLUTION AND CLIMATE-CHANGE MITIGATION

Climate change can enhance the levels of some environmental pollutants, including O_3_ and PM_2.5_. For example, the formation of photochemical smog and O_3_ increases with higher temperatures. Doherty *et al*. quantified the burden of heat and O_3_ on mortality in 15 UK conurbations during the 2003, 2005 and 2006 heatwave periods.^72^ The results indicated that the number of deaths attributable to O_3_ was higher than the number attributable to heat. Furthermore, O_3_ concentrations rose significantly during the summer of 2003, reaching a maximum of 100 ppb.^72^ Ambient concentrations of particles may increase due to forest fires that are a consequence of a dry environment and other climatological effects such as El Niño. During 1998 in Indonesia, forest fires linked with El Niño resulted in the exposure of some 20 million people across South-East Asia to harmful smoke-borne pollutants. Monthly PM_10_ values, which usually fluctuate between 30 and 50 µg/m^3^, increased to between 60 and 110 µg/m^3^ during September–October 1997. The incidence of medical complaints rose by about 30% during this period.

Some of the planned climate-change mitigation strategies include more efficient use of fossil fuels for industrial processes and electricity generation, switching to renewable energy (solar/wind/wave power), increasing the fuel efficiency of vehicles, improving the insulation of buildings, growing new forests, nuclear power and carbon sequestration. It is generally accepted that efforts in all these areas will, at best, prevent further warming but not reverse existing warming.

The Mexican Government recently introduced significant measures aimed at reducing climate change, including a law to reduce carbon dioxide emissions by 30% by 2020 and by 50% below 2000 levels by 2050.^73^ Furthermore, it aims to generate 35% of the country's energy from renewable sources by 2024. At the beginning of 2001, the authorities in Monterrey, Mexico, built a 7-megawatt plant that converts 214 million m^3^ of landfill gas into electricity and powers the light rail transit system and city street lighting at night. Despite these changes, Mexico still faces some hurdles, including enforcement of the new laws; current problems include urban planning, excessive industrialization and traffic jams in large cities. In this regard, the United Kingdom has implemented some measures in London, to take taxis older than 15 years and private hire vehicles older than 10 years off the road, build bicycle superhighways (cycle revolution) and introduce 300 hybrid buses by the end of 2012.

Urban forests and green roofs have also been proposed as strategies for reducing pollution in urban areas.^74^ Vegetation removes pollutants in several ways; by absorbing gaseous pollutants, through interception of PM by leaves, and by breaking down organic compounds such as polycyclic aromatic hydrocarbons.^75^ Transpirational cooling also reduces temperatures indirectly, which results in a reduction in photochemical reactions that form O_3_ and other air pollutants in the atmosphere. It has been estimated that in the United States, trees remove 711 000 metric tonnes of carbon monoxide, nitrogen dioxide, O_3_, PM and sulphur dioxide per year.^76^ However, in many urban areas, there is little space for planting trees or cultivating urban forests. For example, in the mid-Manhattan, west section of New York, 94% of the land is covered with concrete, leaving little space for planting trees at ground level.^77^ However, rooftops, which often comprise nearly half the impermeable area in a city, provide an opportunity for growing vegetation.^78^ Two thousand square metres of uncut grass on a green roof can remove upto 4000 kg of PM.^79^

Public policy and individual action are both required to reduce the effects of pollutants on respiratory health. Interventions at the individual level may include the avoidance of exercise or cycling near busy roadways to reduce exposure, and improvements in the ventilation of homes in which biomass fuels are used. Moreover, public policies can encourage or mandate engineering solutions that drastically reduce emissions from cooking stoves and vehicles. Trials such as those performed during the Beijing Olympics have demonstrated how such changes may have implications for human health. Taken together, these observations suggest that reducing the levels of air pollutants will have a substantial impact on health, particularly the health of patients with respiratory diseases.

The main public health responses to the projected health impacts of climate change are mitigation and adaptation. Adaptation is not an effective risk management strategy for poor air quality, because physiological mechanisms for decreasing susceptibility to O_3_ and other air pollutants are limited. Therefore, if improved modelling experiments continue to predict higher O_3_ concentrations with changing climate, rapid reductions in emissions from the burning of fossil fuels are needed, in order to protect the health of current and future generations. Evidence suggests that reducing current tropospheric O_3_ concentrations reduces morbidity and mortality, with significant savings in the costs of medical care.^80^

## CONCLUSIONS

Air pollution currently affects the health of millions of people. We have presented evidence on the effects of pollutants on patients with limitations in their respiratory capacities. For example, O_3_ and PM may trigger asthma symptoms or lead to premature death, particularly in elderly individuals with pre-existing respiratory or cardiovascular disease. In addition, pollutants enhance the release of allergenic pollen grains, which results in an increased prevalence of pollen-induced asthma. Thus, the case for action to reduce air pollution is overwhelming and this action can take many forms. Some of these include urban planning, technological developments (e.g. the design of new vehicles that produce less pollution), and at the government level, the introduction of new laws. It has been estimated that reducing both black carbon and O_3_ levels would prevent over 3 million premature deaths and increase crop yields by around 50 million tonnes annually. Improvements to cooking stoves would also decrease demand for firewood and reduce deforestation in the developing world. Similarly, improved brick kilns that are used in parts of Latin America and Asia use 50% of the fuel used by traditional kilns.^81^

If air pollution levels in heavy traffic areas were reduced, the incidence of asthma and other respiratory diseases would be significantly reduced.^28^ While it is generally accepted that efforts to reduce air pollution will prevent further environmental changes, they will not reverse existing warming. Interestingly, an increasing number of studies show that in individuals with low anti-oxidant levels, dietary supplements could be used as a promising approach to reducing susceptibility to air pollution, and providing an alternative strategy for neutralizing the effects of pollutants on health.
